# *In silico* mining identifies *IGFBP3* as a novel target of methylation in prostate cancer

**DOI:** 10.1038/sj.bjc.6603767

**Published:** 2007-04-24

**Authors:** A S Perry, B Loftus, R Moroose, T H Lynch, D Hollywood, R W G Watson, K Woodson, M Lawler

**Affiliations:** 1 Department of Haematology and Academic Unit of Clinical and Molecular Oncology, Institute of Molecular Medicine, St James's Hospital and Trinity College Dublin, Ireland; 2 Department of Histopathology, AMNCH and Trinity College Dublin, Ireland; 3 Florida Hospital Cancer Center, Orlando, USA; 4 Department of Urology, St James's Hospital, Ireland; 5 UCD School of Medicine and Medical Science, Conway Institute of Biomolecular and Biomedical Research, University College Dublin, Ireland; 6 Genetics Branch, National Cancer Institute, MD, USA

**Keywords:** methylation, prostate cancer, prostatic intraepithelial neoplasia, insulin-like growth factor binding protein 3, glutathione-*S*-transferase pi

## Abstract

Promoter hypermethylation is central in deregulating gene expression in cancer. Identification of novel methylation targets in specific cancers provides a basis for their use as biomarkers of disease occurrence and progression. We developed an *in silico* strategy to globally identify potential targets of promoter hypermethylation in prostate cancer by screening for 5′ CpG islands in 631 genes that were reported as downregulated in prostate cancer. A virtual archive of 338 potential targets of methylation was produced. One candidate, *IGFBP3*, was selected for investigation, along with *glutathione-S-transferase pi* (*GSTP1*), a well-known methylation target in prostate cancer. Methylation of *IGFBP3* was detected by quantitative methylation-specific PCR in 49/79 primary prostate adenocarcinoma and 7/14 adjacent preinvasive high-grade prostatic intraepithelial neoplasia, but in only 5/37 benign prostatic hyperplasia (*P*<0.0001) and in 0/39 histologically normal adjacent prostate tissue, which implies that methylation of *IGFBP3* may be involved in the early stages of prostate cancer development. Hypermethylation of *IGFBP3* was only detected in samples that also demonstrated methylation of *GSTP1* and was also correlated with Gleason score ⩾7 (*P*=0.01), indicating that it has potential as a prognostic marker. In addition, pharmacological demethylation induced strong expression of *IGFBP3* in LNCaP prostate cancer cells. Our concept of a methylation candidate gene bank was successful in identifying a novel target of frequent hypermethylation in early-stage prostate cancer. Evaluation of further relevant genes could contribute towards a methylation signature of this disease.

Prostate cancer is the most common noncutaneous malignancy and a leading cause of cancer-related deaths in men in the Western world (Jemal *et al*, 2006). Although gland-confined disease is potentially curable, the inevitable emergence of androgen insensitivity in late-stage tumours is ultimately fatal. The molecular events leading to the initiation and development of prostate cancer are not well understood. High-throughout quantitative transcriptomic studies have revealed large numbers of gene expression changes (Dhanasekaran *et al*, 2001; Ashida *et al*, 2004), which suggest that a broad and complex network of molecular alterations is involved. In addition to deciphering the identities and functions of these genes, it is important to address the mechanisms that govern their deregulation.

The integral role of epigenetic mechanisms such as promoter hypermethylation in the silencing of tumour suppressor genes has become ever more apparent over the past decade (Herman and Baylin, 2003). Promoter hypermethylation is widespread in prostate cancer; described at early stages of cancer development in preinvasive high-grade prostatic intraepithelial neoplasia (HGPIN) and has been correlated with clinicopathologic features indicative of a poor prognosis, indicating the potential of gene hypermethylation as a marker of clinically significant disease (Perry *et al*, 2006).

The most promising methylation biomarker identified to date is *glutathione-S-transferase pi* (*GSTP1*), detected in >90% of prostate tumours, >70% of HGPIN and at significantly lower frequencies and quantitatively much lower levels in normal prostate and benign prostatic hyperplasia (BPH) (Nakayama *et al*, 2003; Jeronimo *et al*, 2004). In addition, methylation of *GSTP1* is highly specific to prostate cancer, rarely detected in other tumours (Esteller *et al*, 2001). *Glutathione-S-transferase pi* is methylated throughout all stages and grades of prostate cancer. Therefore, the efficacy of *GSTP1* as a prognostic biomarker of clinically significant prostate cancer would undoubtedly be improved with the discovery of further epigenetic targets in this disease. Identifying novel targets of hypermethylation from transcriptional data repositories may also shed insight into potential pathways of this disease, as well as highlight individual genes relevant to prostate carcinogenesis.

We developed an *in silico* strategy to globally identify potential targets of promoter hypermethylation in prostate cancer. This approach yielded a database of over 300 potential targets of methylation in prostate cancer. Several lines of evidence supported an investigation into the *Insulin-like growth factor binding protein 3* (*IGFBP3*) gene. IGFBP3 is the most abundant IGF-binding protein in the circulation, where it controls the half-life and bioavailability of IGF1 for signal transduction through the IGF1 Receptor (IGF1R), which leads to the activation of growth promoting pathways, including the Ras/Raf/MAP kinase and the phosphotidylinositol-3 kinase pathways (Renehan *et al*, 2004; Papatsoris *et al*, 2005). Overexpression of IGFBP3 has dramatic growth-inhibitory and proapoptotic effects in murine prostate tumours and prostate cancer cell lines (Modric *et al*, 2001; Devi *et al*, 2002), indicating that IGFBP3 may act as a tumour suppressor in prostate cells. IGFBP3 also exerts a variety of IGF-independent proapoptotic and antiproliferative effects, shown by the finding that IGFBP3 overexpression inhibits the growth of fibroblasts that are IGF1R negative (Oh *et al*, 1993).

Promoter hypermethylation has been proposed as a mechanism responsible for transcriptional silencing of *IGFBP3* in hepatocellular carcinoma (Hanafusa *et al*, 2002), non-small cell lung carcinoma (Chang *et al*, 2002) and very recently in cancers of the bladder and ovary (Christoph *et al*, 2006; Wiley *et al*, 2006). In this study, we performed an extensive analysis of the methylation pattern of *IGFBP3* in benign, preinvasive and cancerous prostate tissues. Concordance between *IGFBP3* and *GSTP1* methylation was investigated. Statistical analysis was performed to investigate a correlation between methylation and clinical and pathologic parameters. In addition, prostate cancer cell lines were employed to test whether promoter hypermethylation affects *IGFBP3* gene expression.

## MATERIALS AND METHODS

### *In silico* mining to identify novel targets of methylation in prostate cancer

We developed an *in silico* approach to identify potential targets of promoter hypermethylation in prostate cancer. The principle behind this concept was that promoter hypermethylation leads to a reduction in gene expression at the transcriptional level. Two freely available web-based transcriptome databases were employed to identify genes downregulated in prostate cancer compared with normal prostate. Nine queries were performed on the Gene Expression Atlas (http://expression.gnf.org) to retrieve genes that were both expressed in normal prostate >1 times – and in prostate cancer <0.5–1 times – the median expression across 46 human tissues (Su *et al*, 2001). The Digital Differential Display (http://www.ncbi.nlm.nih.gov/UniGene/ddd) was used to report on significant differences in gene expression between four different tissue pools, created from libraries of ESTs from normal prostate, primary and metastatic prostate cancer and PIN (Wheeler *et al*, 2001). The data from four independent, published microarray studies that quantified gene expression at different stages of prostate cancer were also examined (Bubendorf *et al*, 1999; Chetcuti *et al*, 2001; Dhanasekaran *et al*, 2001; Ashida *et al*, 2004).

Each gene was screened for the presence of a 5′ CpG island using the UCSC Human Genome Browser (http://genome.ucsc.edu/) to positively filter for genes that could be susceptible to promoter hypermethylation (Kent *et al*, 2002). Successful targets were organised by putative function using GeneCards (http://genecards.org) (Rebhan *et al*, 1997). CpG islands were characterised for promoter sequences using Promoter Scan (http://thr.cit.nih.gov/molbio/proscan/) (Prestridge, 1995).

### Clinical sample collection

Prostate tumours and histologically normal adjacent tissue from 40 patients with primary disease, treated by radical prostatectomy at The Adelaide and Meath Hospital (AMNCH), Dublin, Ireland, were obtained retrospectively. Histologically normal adjacent tissue was tissue not involved by prostate cancer, HGPIN or BPH. High-grade prostatic intraepithelial neoplasia lesions were obtained from 14 cases. For control purposes, BPH lesions from 37 men without prostate cancer that underwent transurethral resection (TURP) of the prostate were also collected. Additionally, a further 39 primary tumours were obtained from the Florida Hospital Cancer Institute, USA (FHCI) as part of a collaboration with the National Cancer Institute (NCI), USA. The Gleason score, TNM classification (Fleming *et al*, 1997), PSA level and age at diagnosis were obtained from the relevant clinical records. This study was approved by the AMNCH and St James's Hospital Ethics committee. The FHCI specimens were approved for research purposes by the NCI Office of Human Subjects Research.

Histological slides from the formalin-fixed, paraffin-embedded (FFPE) surgical specimens were reviewed by a pathologist to identify areas of histologically normal prostate, prostate cancer, HGPIN and BPH. A series of 5 *μ*m sections were cut from the FFPE blocks. The first tissue section from each block was hematoxylin and eosin stained and compared with the pathologically evaluated slide, to ensure a consistent percentage of target cells. The target cell populations were the glandular epithelial cells. Adjacent sections were deparaffinised in xylene and rehydrated in decreasing concentrations of ethanol. Tissue was scraped from within the pathologically marked areas using a sterile blade and DNA was extracted using the QIAamp DNA micro kit (Qiagen, Crawley, UK).

### Cell culture and drug treatment

Human prostate cancer cell lines LNCaP, DU145 and PC-3 and normal prostate cell line PWR-1E were propagated under standard cell culture conditions. The effect of DNA methylation on *IGFBP3* expression was measured in LNCaP cells by treatment with the demethylating agent 5-azacytidine. LNCaP cells (2 × 10^6^) were seeded in 75 cm^3^ culture flasks. Twenty-four hours after plating, cells were treated with 2 *μ*M 5-azacytidine (Sigma, Dublin, Ireland) and the medium was changed after 24 h. After 2 days of treatment, cells were harvested. DNA was isolated from cell lines by use of a QIAamp DNA mini kit (Qiagen, UK) and total RNA was extracted using the Trizol method (Invitrogen, Paisley, UK).

### Bisulfite modification and quantitative methylation specific PCR

Sodium bisulphite modification of genomic DNA converts unmethylated (but not methylated) cytosines to uracils. Bisulfite modification of approximately 20 ng of DNA isolated from tissue samples and up to 1 *μ*g of cell line DNA, was performed with the EZ DNA methylation kit (Zymo Research, Orange, CA, USA). Modified DNA was eluted into a final volume of 50 *μ*l 1 × Tris EDTA (Sigma, Ireland).

Methylation was evaluated by real time quantitative methylation specific PCR (QMSP) as described by Eads *et al* (2000). Parallel TaqMan PCR reactions were performed on every sample with oligonucleotides targeted to an (i) endogenous control gene (*β-actin*) for unbiased amplification of bisulphite modified DNA and (ii) target genes (*GSTP1* and *IGFBP3*) for amplification of bisulphite modified fully methylated molecules (Table 1). A quantity of product was obtained for each reaction by interpolating from a standard curve, constructed with 10-fold serial dilutions of bisulfite modified, universal methylated DNA (Chemicon International, Temecula, CA, USA). The level of methylated target in each sample was then determined by a relative methylation score (RMS), by applying the formula: (*target* quantity/*β-actin* quantity) × 1000.

All assays were performed in duplicate on a 7900 HT Sequence Detection System (Perkin-Elmer, Foster City, CA, USA), in a final volume of 10 *μ*l, which consisted of 2 *μ*l bisulfite-modified DNA, primers (MWG Biotech, Ebersberg, Germany), fluorescent labelled probe (Applied Biosystems, Warrington, UK) and 5 × TaqMan Universal PCR master mix, no AmpErase Uracil N-Glycosylase (Roche, Branchburg, NJ, USA), under standard TaqMan real-time PCR cycling conditions. Samples were considered positively amplified when a comparative threshold cycle (*C*_T_) <50 was detected in both duplicates, with <1 *C*_T_ variance between duplicates.

Methylation of *IGFBP3* was investigated by three QMSP assays designed to evaluate CpG dinucleotides at different locations (promoter and coding sequence) within the promoter CpG island: *IGFBP3-A*: −406 to −328, *IGFBP3-B*: −217 to −150 and *IGFBP3-C*: +132 to +228. Altogether, the primer and probe sets covered 30/139 CpG sites throughout the region, spanning more than 600 bp of the island.

### Quantitative reverse transcription PCR

cDNA was synthesised from 1 *μ*g RNA using M-MLV reverse transcriptase and random hexamers (Invitrogen, UK) at 37°C for 1 h in a total volume of 30 *μ*l. A primer and probe set was targeted to exons 2 and 3 of the *IGFBP3* gene, to avoid amplification of DNA, forward primer 5′-agtccaagcgggagacagaa-3′, reverse primer 5′-caggtgattcagtgtgtcttcca-3′, probe 6FAM5′-ccctgccgtaga gaa-3′MGB. cDNA (100 ng) was used as a template for real-time TaqMan reactions, as described above. The levels of *IGFBP3* expression in cell lines were calculated as a fold change from a calibrator sample (normal prostate cell line PWR-1E) using the Comparative *C*_T_ method by applying the formula 2^−ΔΔ*C*_T_^ (Livak and Schmittgen, 2001). The amount of IGFBP3 in each sample was normalised to an endogenous reference gene, using a *β*-actin pre-developed assay (Applied Biosystems, UK).

### Statistical analysis

Statistical analysis was performed using MINITAB (version 1.4). Differences in frequencies of methylation between histologically normal, BPH, HGPIN and prostate cancer were assessed using Fisher's exact Test. Differences in methylation levels of genes were compared by examining the RMSs between samples using the Kruskal–Wallis one-way ANOVA test and the Wilcoxon-matched pairs test. An Unpaired *t*-test was used to calculate the differences in age and PSA between patient groups. For all of the tests, significance was ascribed at *P*<0.05.

## RESULTS

### Identification of potential targets of methylation in prostate cancer

A list of 631 (known and unknown) genes that are downregulated in prostate cancer was generated through our *in silico* strategy. Of these genes, 355 (56.26%) possessed a CpG island within 5 kb +/− of their transcriptional start site (Supplementary Table 1). 16/355 genes were commonly identified as downregulated in more than one study (*ATF3*, *CAV2*, *CCND2*, *CTSB*, *CYP1B1*, *EPB72*, *GSTM1*, *ID2*, *IGFBP3*, *MGFE8*, *NET1A*, *OAT*, *RPS19*, *SGK*, *SHB* and *ZFP36*). The appearance of known targets of methylation, *CCND2*, *CD44*, *ECAD* and *GADD45A*, demonstrated the success of this technique in identifying (potential) targets of methylation in prostate cancer. We selected *IGFBP3* as our first target to evaluate.

### Clinical characteristics

Methylation was investigated in tissue samples of prostate cancer, histologically normal prostate, HGPIN and BPH. As expected, PSA levels were higher in patients with prostate cancer (9.74 ng ml) than with BPH (6.39 ng ml^−1^) (*P*=0.018, 95% CI=1.15, 3.72), although there was considerable overlap in the range of measures. Notably, the mean PSA of the BPH patients was above 4.0 ng ml^−1^, the widely accepted upper limit of normal. The difference in mean age between the prostate cancer (61.67 years) and BPH (75.41 years) groups was statistically significant (*P*<0.0001, 95% CI=10.50, 16.97).

### *IGFBP3* methylation in prostate tissues

**Figure 1:**
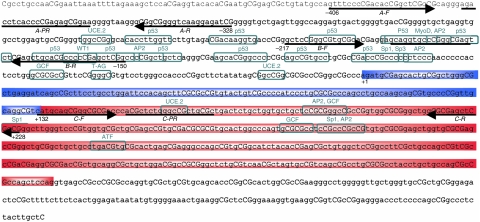
Genomic sequence analysis of *IGFBP3* (GenBank accession number M35878). The 1.2 kb IGFBP3 CpG island begins 476 bp upstream of the 5′ untranslated region (shown in blue), extends through the first exon (shown in red) and terminates 199 bp into the first intron. Putative transcription factor binding sites that contain CpG sites are indicated. Primer and probe sequences for QMSP assays (**A**, **B** and **C**) are shown with black arrows and lines, respectively.

**Figure 2:**
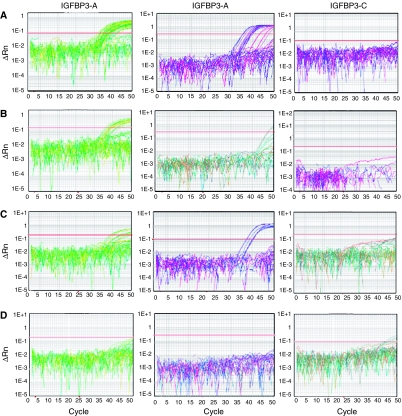
Differential methylation of *IGFBP3* in prostate cancer. QMSP amplification plots in (**A**) prostate cancer, (**B**) HGPIN, (**C**) BPH and (**D**) histologically normal adjacent prostate samples. Methylation was only detected in two of the morphologically normal samples at *IGFBP3-C* at cycle number 43, indicating very low levels of methylation in these samples.

The characterised promoter CpG island of *IGFBP3* is shown in Figure 1. *IGFBP3* methylation was detected in 49/79 (62.03%) of the prostate cancer samples at sequence A, 15/79 (19.23%) at sequence B and 0/79 (0%) at sequence C (Figure 2).

*IGFBP3* was completely unmethylated in the histologically normal prostate samples, except for two cases that contained methylated DNA in the *IGFBP3-C* region. In the BPH samples, the frequencies of methylation were very low: *IGFBP3-A*, 5/37 (13.51%); *IGFBP3-B*, 2/37 (5.41%) and *IGFBP3-C* 0/37 (0%). In total, 7/37 (18.9%) of the hyperplastic prostates showed methylation of *IGFBP3*, a frequency significantly less than detected in the tumours (*P*<0.0001). There was no significant difference in the methylation frequency between the histologically normal and BPH groups.

HGPIN lesions were obtained from 14/79 patients with prostate cancer. *IGFBP3* methylation was only detected in HGPIN samples from patients whose adjacent tumour was also methylated. *IGFBP3-A* methylation was found in 7/14 (50%) cases, *IGFBP3-B* in 1/14 (7.14%) cases and *IGFBP3-C* in 0/14 cases. The frequency of *IGFBP3* methylation in HGPIN was not statistically different from tumour (*P*=0.383).

### Methylation of *GSTP1* and *IGFBP3*

Hypermethylation of *GSTP1* was identified in 75/79 (94.94%) tumours, 10/14 (71.43%) HGPIN, 5/39 (12.82%) histologically normal prostate samples and 4/37 (10.81%) BPH. Statistically significant differences were found for the methylation status of *GSTP1* between prostate cancer and both BPH and histologically normal adjacent tissues (*P*<0.0001). In all 49 tumours and 6/7 HGPIN that showed *IGFBP3* methylation, methylation of *GSTP1* was also detected. Methylation of *IGFBP3* was only detected in those samples (both tumour and the majority of HGPIN) that demonstrated *GSTP1* methylation. Notably, methylation of both genes was not detected in any of the histologically normal or BPH samples.

### Methylation levels in prostate tissues

**Figure 3:**
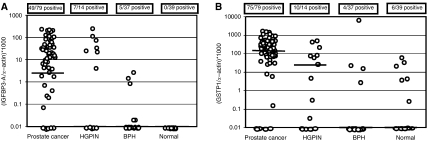
Distribution of (**A**) *IGFBP3-A* and (**B**) *GSTP1* methylation levels in prostate cancer, HGPIN, normal prostate and BPH. The median RMS is indicated by a horizontal line. Values diagrammed at 0.01 are zero values, which could not be plotted correctly on a log scale.

In addition to determining the frequencies of *IGFBP3* and *GSTP1* methylation, the levels of methylation were compared between the different sample types (Figure 3). Although there was a significant difference between the methylation levels of *GSTP1* and *IGFBP3* within tumour samples (*P*<0.0001; 95% CI=88.17, 174.22), the median RMS of both genes was significantly higher in tumours than in histologically normal adjacent prostate or BPH (*P*<0.0001).

### Promoter hypermethylation and clinicopathologic correlations

Results for correlations of the methylation status of *IGFBP3* and *GSTP1* with clinicopathologic factors (Gleason score, TNM classification and PSA level) are shown in Table 2. Methylation of *GSTP1* was not significantly associated with any pathological parameters. Methylation of *IGFBP3* was detected in significantly more tumours with Gleason score ⩾7, than ⩽6 (*P*=0.01), but was not significantly correlated with TNM classification or PSA level.

### IGFBP3 methylation and gene silencing in prostate cancer cell lines

**Figure 4:**
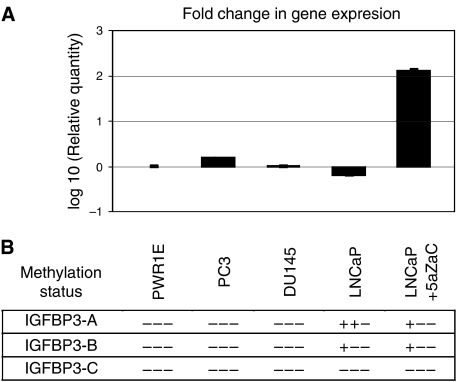
*IGFBP3* methylation and expression analyses in prostate cancer cell lines. (**A**) The fold change in mRNA expression of *IGFBP3* in prostate cancer cell lines relative to normal prostate cell line PWR1E. QRT–PCR showed that all cell lines expressed *IGFBP3* mRNA but the LNCaP cell line showed ∼3-fold reduction in expression. This was reversed upon treatment with demethylating drug 5-azacytidine. (**B**) The relative level of *IGFBP3* methylation in cell lines was examined by QMSP and is displayed by +++: 1000, ++−: 999–500, --+: 499–1 and ---: 0.

The methylation pattern of *IGFBP3* was examined in a panel of prostate cancer cell lines (Figure 4). Only the LNCaP cell line was found to possess methylated *IGFBP3* alleles by amplification with the A and B primer sets. The demethylation of *IGFBP3* by 5-azacytidine in this cell line was confirmed by QMSP. QRT–PCR revealed that the three cancer cell lines expressed *IGFBP3* relative to the normal cell line PWR-1E, however, pharmacological demethylation of LNCaP increased the expression of *IGFBP3* several hundred-fold.

## DISCUSSION

Recognition of the importance of promoter hypermethylation in human cancer has fostered a growing effort to screen the cancer genome to identify methylated loci. In this study, we describe a novel *in silico* approach to identify potential targets of methylation in prostate cancer. We employed transcriptomic databases and microarray experiments that provided detailed data summaries and focused on expression changes in early-stage disease (Chetcuti *et al*, 2001; Ashida *et al*, 2004) and in the progression to metastatic disease (Bubendorf *et al*, 1999; Dhanasekaran *et al*, 2001). Interestingly, a common finding between these studies was that downregulation rather than upregulation, accounted for the majority of differentially expressed genes in prostate cancer. This suggests that the full force of transcriptional silencing mechanisms in prostate cancer may not yet be fully recognised.

Approximately half of the genes examined contained a 5′ CpG island, which was consistent with previous reports (Takai and Jones, 2002; Wang and Leung, 2004). To select the most interesting gene(s) for methylation analysis from over 300 candidates required further validation, beyond the identification of a promoter CpG island. Furthermore, we recognised that there are substantial challenges in the interpretation of data obtained from large-scale gene expression arrays. For example, which of the hundreds of differentially expressed genes are important primary events and which are downstream or secondary events? To maximise the likelihood of choosing the most relevant genes for analysis, literature review was performed on all of the candidates. This led to the selection of the *IGFBP3* gene, whose methylation status has not been previously reported on in prostate cancer.

A detailed map of the *IGFBP3* promoter CpG island facilitated QMSP assay design around functionally relevant regions of the island. Using three QMSP assays, we found tumour-related differential methylation of the *IGFBP3* promoter CpG island in prostate cancer. Methylation was exclusive to the non-coding part of the island. The high frequency (62%) observed at the 5′ end of the island (assay-A) decreased to 19% around the MyoD, AP-2, p53 and WT-1 transcription factor binding sites (assay-B) and diminished to zero at the start of the first exon (assay-C). Of the 15 tumours methylated at assay-B, all but one were also methylated at the upstream part of the island, which supports the theory that *de novo* hypermethylation may gradually propagate through an island during neoplastic progression, initiating from the outer flanks of the CpG island (Mummaneni *et al*, 1995; Graff *et al*, 1997). An interesting finding was that the majority of prostate cancers were unmethylated around a methylation hotspot (corresponding to the p-53, AP-2 and Sp-1/Sp3 binding sites), reported in both non-small cell lung carcinoma (61%) and hepatocellular carcinoma (33%) (Chang *et al*, 2002; Hanafusa *et al*, 2002). These results clearly show that methylation is not uniformly spread throughout the *IGFBP3* CpG island and therefore, the choice of CpG sites for investigation is an important consideration.

To address whether *IGFBP3* methylation is involved in the early stages of disease initiation, we examined the relationship between methylation of *IGFBP3* in prostate cancer and HGPIN isolated from the same prostatectomy samples. Methylation was only detected in those HGPIN lesions, whose adjacent tumour sample from the same patient was also methylated. This implies that hypermethylation of *IGFBP3* occurs as an early event in prostate carcinogenesis and supports a clonal relation between the tumour and HGPIN lesion. We also found methylation of *GSTP1* in a high frequency of HGPIN foci, consistent with previous reports (Nakayama *et al*, 2003; Kang *et al*, 2004).

Although the frequency and levels of *GSTP1* methylation were significantly higher than *IGFBP3* methylation, an unexpected finding was the concordance between methylation of both genes across all tumours and all but one HGPIN. The high prevalence of *GSTP1* hypermethylation in prostate cancer, HGPIN and in some proliferative inflammatory atrophy lesions indicates that it most likely precedes many other molecular aberrations in prostate carcinogenesis (Nakayama *et al*, 2003). Associated loss of GSTP1 activity and its protection from electrophilic and oxidative DNA damage would render cells susceptible to further transformations. Promoter hypermethylation of *IGFBP3* likely occurs as a subsequent epigenomic ‘hit’ in the multi-step process of cancer development.

Low levels of methylation were detected for both *IGFBP3* and *GSTP1* in a small percentage of benign prostate samples. Consistent with our results, other studies have also described quantitatively much less promoter methylation of *GSTP1* in BPH and histologically normal tissue (from tumour containing prostates) than in cancer specimens (Jeronimo *et al*, 2001; Yamanaka *et al*, 2003). Although every effort was taken to ensure that tissue was only procured from within pathologically identified areas, we cannot rule out the possibility that minute amounts of occult carcinoma or HGPIN may have been present in some of the benign specimens. However, even studies that employed laser capture microdissection to isolate pure populations of cells have reported low levels of hypermethylation in morphologically normal prostate samples (Henrique *et al*, 2006). An alternative explanation is that certain molecular aberrations such as promoter hypermethylation may precede morphological changes, initially affecting a small subset of benign epithelial cells. In support of this hypothesis, none of the benign samples demonstrated any evidence of methylation spreading, that is, they were not that amplifiable with primer and probe sets *IGFBP3-A* and *IGFBP3-B*.

Reduced expression of IGFBP3 in the prostate epithelium is correlated with cancer progression from HGPIN to localised cancer and androgen-independent disease (Thrasher *et al*, 1996; Hampel *et al*, 1998; Nickerson *et al*, 2001). Rising PSA levels during the course of prostate cancer progression may facilitate tumorigenesis by digesting IGFBP3 and releasing free IGF-1 into the prostate microenvironment (Cohen *et al*, 1992). However, this post-translational proteolysis cannot account for reduced *IGFBP3* mRNA levels (Ashida *et al*, 2004; Thelen *et al*, 2004). Examination of *IGFBP3* expression *in vitro*, revealed that all of the prostate cancer cell lines expressed *IGFBP3*, including LNCaP cells whose methylation pattern was representative of the majority of prostate tumours analysed (heavily methylated at *IGFBP3-A*, remarkably fewer CpGs methylated at *IGFBP3-B* and unmethylated at *IGFBP3-C*). However, 5-azacytidine treatment of LNCaP reversed the hypermethylation pattern of *IGFBP3* and resulted in a significant induction in expression. Although these findings could indicate monoallelic methylation in LNCaP, previous reports would suggest that expression is maintained because methylation is not affecting core promoter sequences (Chang *et al*, 2004; Hanafusa *et al*, 2005). Whether methylation spreading throughout the island in approximately 20% of prostate cancers correlates with a reduction in *IGFBP3* expression, warrants further investigation.

With a shift toward earlier stages at diagnosis because of PSA screening, it is becoming increasingly important to understand the aetiology of prostate cancer recurrence and progression. Identifying methylation events early in carcinogenesis that are correlated with potentially aggressive tumours could identify those patients more likely to recur and avoid the over-treatment of those indolent tumours that may otherwise never require therapy. In support of previous studies, our data show methylation of *GSTP1* is highly prevalent across all grades and stages of disease (Jeronimo *et al*, 2001; Yegnasubramanian *et al*, 2004). However, methylation of *IGFBP3* was associated with advanced tumour grade. Increased density of methylation within a CpG island has been associated with more advanced stages of tumours (Nosaka *et al*, 2000). Although we did not detect a statistically significant relationship between tumour samples amplified by both primer sets *IGFBP3-A* and *IGFBP3-B* and Gleason score (*P*=0.09), this may be attributable in part to the small sample size. Non-small cell lung carcinoma patients with methylation of *IGFBP3* had significantly poorer overall survival probability compared with those without methylation (Chang *et al*, 2002). In addition, methylation levels of *IGFBP3* have been associated with tumour recurrence in transitional cell carcinoma of the bladder (Christoph *et al*, 2006). The results from this study indicate that such an investigation is warranted in prostate cancer.

In summary, our concept of *in silico* data mining was successful in identifying a novel target of frequent methylation in prostate cancer and HGPIN. Our findings also emphasise the importance of thorough investigations into methylation patterns in order to gain an insight into the dynamics of *de novo* hypermethylation and its relationship to transcriptional silencing. Although *GSTP1* is widely recognised as an excellent biomarker of prostate cancer, used alone it has limitations. The inherent clinical and genetic heterogenic nature of prostate cancer suggests that profiling the cumulative methylation of multiple genes would serve to better distinguish benign from malignant tissues and would provide a more powerful approach in the early detection of prostate cancer and in identifying those men who should be targeted for more aggressive therapy, than characterising the status of only one gene marker. We are screening further potential targets of methylation that we have identified to contribute towards a methylation fingerprint of this disease.

## Figures and Tables

**Table 1:** QMSP primer and probe sequences

	**Primer set (5′–3′) forward, reverse**	**Probe (6FAM5′-3′MGB)**	**Primer, probe (nM)**
*β*-actin	ggt gga ggt agt tag ggt tta ttt gta	cac ttt tat tca act aat ctc	300, 100
	cca cac cac aaa atc aca ctt aac ctc att t		
IGFBP3-A	ttt ttt cga tat cgg ttc gtc g	aga ttt tat ttc gag agc gga	300, 100
	gat ctc ctt aac ccc gcc g		
IGFBP3-B	ggt ttc ggg cgt gcg tac	tag gtg ttc gcg cga gtt t	300, 100
	ccg aac tcg aaa acg tac aac tcg		
IGFBP3-C	Tat gta gcg ggc gcg att	cgt ttt ggg tcg ttg cg	900, 300
	cgc cga act cgc gc		
GSTP1	gtt gcg tgg cga ttt cg	cga cga ccg cta cac	300, 300
	cga act ccc gcc gtc c		

**Table 2:** Frequency of *IGFBP3* and *GSTP1* methylation in histologically normal and cancerous prostate tissue, HGPIN and BPH, and relationship to clinicopathologic factors

	**IGFBP3-A *n* (%)**	**IGFBP3-B *n* (%)**	**IGFBP3-C *n* (%)**	**GSTP1 n (%)**
Normal prostate	0/39 (0)	0/39 (0)	2/39 (5.3)	5/39 (12.82)
BPH	5/37 (13.51)	2/37 (5.41)	0/37 (0)	4/37 (10.81)
HGPIN	7/14 (50)	1/14 (7.14)	0/14 (0)	10/14 (71.43)
Prostate cancer	49/79 (62.03)	15/79 (19.23)	0/79 (0)	75/79 (94.94)
				
*Gleason score*				
⩽6	17/37 (45.95)	4/37 (10.81)		35/37 (94.59)
⩾7	32/42 (76.19)	11/42 (26.19)		40/42 (95.24)
*P*-value	0.01	0.09		1
				
*TNM classification*				
pT2	35/55 (63.64)	11/55 (20)		51/55 (94.44)
pT3, pT4	12/20 (60)	3/20 (15)		20/20 (100)
*P*-value	0.8	0.8		1
				
*PSA (ng ml)*				
<4	5/9 (55.56)	1/9 (11.11)		8/9 (88.89)
4–10	27/43 (62.79)	6/43 (13.95)		42/43 (97.67)
>10	13/22 (59.1)	6/22 (27.27)		20/22 (90.91)
*P*-value	0.9	0.37		0.38
