# Supplementary material for: In silico mining identifies IGFBP3 as a novel target of methylation in prostate cancer
Source: Br J Cancer. 2007 Apr 24;96(10):1587–94. doi: 10.1038/sj.bjc.6603767 (PMC2359953; doi:10.1038/sj.bjc.6603767)
Supplement: Supplementary file 1 [file 6603767x1.doc]

SUPPLEMENTAL TABLE 1 Potential targets of methylation in prostate cancer

Gene Symbol[†]	GenBank Accession number	Gene Description	GEA	DDD	Microarray studies				
					1	2	3	4	5
Apoptosis									
BCL2	M14745	B cell CLL / lymphoma 2	Y						
CFLAR	NM_003879	CASP8 and FADD-like apoptosis regulator				Y			
CLU	BC010514	Clusterin			Y				
SMAD4	NM_005359	Mothers against DPP homolog 4							
DAP	NM_004394	Death associated protein				Y			
RAGA	NM_006570	Ras-related GTP binding protein				Y			
IER3	S81914	Immediate early response 3						Y	
PIGPC1	AA253194	p53-induced protein PIGPC1							Y
SPHK2	AA485440	Sphinosine Kinase 2	Y						
Regulation of Cell Cycle									
CCND2	X68452	Cyclin D2	Y			Y			Y
CCNC	NM_005190	Cyclin c isoform A				Y			
CCNH	NM_001239	Cyclin H			Y				
WEE1	NM_003390	Wee1& S. pombe homologue				Y			
CCNG2	NM_004354	Cyclin G2				Y			
PPP1CB	NM_002729	Protein phosphatase 1, catalytic subunit, β isoform				Y			
CCT4	NM_006430	Chaperonin containing TCP1, subunit 4				Y			
CDC42	NM_001791	Cell division cycle 42				Y			
CDC14	AI750036	Cell division cycle 14 homolog B							Y
TGFB1	NM_000660	Transforming growth factor , beta 1				Y			
TGFB2	NM_003238	Transforming growth factor, beta 2				Y			
MAPK6	NM_002748	Mitogen activated protein kinase 6				Y			
chromosomal/DNA binding									
TOP2A	L47276	Topoisomerase II alpha	Y						
PCM1	NM_001697	Pericentriolar material 1				Y			
TOP2B	U54831	Topoisomerase (DNA) IIbeta 180kDa						Y	
H2BFQ	NM_003528	H2B histone family, member Q				Y			
G22P1	NM_00146	Thyroid autoantigen 70kD (ku antigen)				Y			
SMARCD3	AI423028	SWI/SNF related, matrix associated, actin dependent						Y	
LMNA	X03444	Laminin A/C							Y
ZFP36	NM_003407	Zinc finger protein homologous to Zfp-36 in mouse				Y			Y
Immune Response									
MST1	NM_020998	Macrophage stimulating 1				Y			
HLA-F	NM_018950	Major histocompatibilty complex, class I, F							Y
LEF1	AL049409	Lymphoid enhancer binding factor 1	Y						
TCL1A	X82240	T-cell leukaemia/lymphoma 1A	Y						
FBLN1	X53742	Fibulin 1	Y						
CNIL	NM_005776	Cornichon-like				Y			
MCP	NM_002389	Membrane cofactor protein				Y			
HLA-DPB1	NM_002121	Major histocompatibility complex class II DP βI				Y			
CR2	M26004	Complement component CD21	Y						
Nuclease									
RNASE4	NM_002937	Ribonuclease, RNase A family 4				Y			

SUPPLEMENTAL TABLE 1 *continued*

Gene Symbol[†]	GenBank Accession number	Gene Description	GEA	DDD	Microarray studies				
					1	2	3	4	5
**Cell adhesion/motility/cytoskeleton/structural**									
PLAU	D00244	Plasminogen activator, urkinase							Y
FLNA	NM_001456	Filamin A, alpha						Y	
ADD2	U43959	Adducin 2 beta	Y						
FER1L3	AF182316	Fer-1-like 3, myoferlin (C. elegans)							Y
GPC1	NM_002081	Glypican 1						Y	
CSPG2	U16306	Chondrotin sulfate proteoglycan 2						Y	
LAMA4	NM_002290	Laminin, alpha 4				Y			
CTNNB1	NM_001904	Catenin β1				Y			
COL7A1	L02870	Collagen, type VII, alpha 1						Y	
CD164	NM_006016	Sialomucin CD164				Y			
CDH3	X63629	Cadherin 3, type 1 (placental)						Y	
CDH1	NM_004360	Cadherin 1, E-cadherin (epithelial)				Y			
SPINT2	NM_021102	Serine protease inhibitor, kunitz type, 2				Y			
CAPZA2	NM_006136	Capping protein muscle Z line α2				Y			
PAK1	NM_002576	p21/Cdc42/Rac1-activated kinase 1							Y
IQGAP1	NM_003870	IQ motig containing GTPase activating protein 1				Y			
THBS1	NM_003246	Thrombospondin 1				Y			
MRLC2	NM_033546	mRNA for myosin regulatory light chain				Y			
MYPT1	NM_002480	Myosin phosphatase, target subunit 1				Y			
MYL9	J02854	Myosin, light polypeptide 9, regulatory						Y	
MAP7	NM_003980	Microtubule associated protein 7							
HINT	NM_005340	Histidine triad nucleotide-binding protein				Y			
FN1	NM_002026	Fibronectin 1				Y			
SMTN	NM_006932	Smoothelin				Y			
PTPRF	NM_002840	Protein tyrosine phosphatase, receptor type F				Y			
TSPAN-3	NM_005724	Tetraspan 3				Y			
RSN	NM_002956	Restin isoform a				Y			
EPB72	NM_004099	Erythrocyte membrane protein band 7.2				Y			Y
CD44	NM_000610	CD44 antigen				Y			
CTGF	NM_001901	Connective tissue growth factor				Y			
COL15A1	NM_001855	Collagen, type XV, alpha 1				Y			
KRT7	X03212	Keratin 7						Y	
COL6A1	X15880	Collagen, type VI, alpha 1							Y
KIAA0921	NM_015080	KIAA0921 protein (neurexin 2)				Y			
GSN	NM_000177	Gelsolin (amyloidosis, Finnish type)				Y			
VIM	NM_003380	Vimentin				Y			
KRT8	NM_002273	Keratin 8				Y			
FAT	NM_005245	FAT tumour suppressor (Drosophila) homologue				Y			
MUC1	J05581	Mucin 1, transmembrane						Y	
ITGB4	X52186	Integrin beta 4						Y	
ITGB1	NM_002211	Human beta-1D integrin				Y			
TJP1	L14837	Tight junction protein	Y						
ANXA2	D00017	Annexin A2						Y	
ANXA7	NM_001156	Annexin 7				Y			
ANXA11	NM_145869	Annexin 11				Y			

SUPPLEMENTAL TABLE 1 ***continued***

Gene Symbol[†]	GenBank Accession number	Gene Description	GEA	DDD	Microarray studies				
					1	2	3	4	5
Development									
FZD7	NM_003507	Frizzled (Drosophila) homologue 7				Y			
FHL1	NM_001449	Four and a half LIM domains 1				Y			
ID-1	D13889	DNA binding protein inhibitor 1					Y		
Gene Expression									
FOXO1A	NM_002015	Forkhead box 01A				Y			
NR2F1	NM_005654	Nuclear receptor subfamily 2, group F, member 1				Y			
JUND	NM_005354	Jun D proto-oncogene				Y			
JUN	NM_00228	V-jun avian sarcoma virus 17 oncogene homologue				Y			
NET1A	NM_005863	Guanine nucleotide regulatory protein	Y			Y			
CUTL1	L12579	Cut-like 1 CCAAT displacement protein (Drosophila)						Y	
GATA3	AW949747	GATA binding protein 3						Y	
NCOA7	NM_181712	Nuclear receptor coactivator 7						Y	
HOXD11	AA490691	Homeo box D11						Y	
RFC3	NM_002915	Replication factor C (activator 1) 3				Y			
NR4A3	NM_006981	Nuclear receptor subfamily 4, group A, member 3				Y			
TCF12	NM_003205	Transcription factor 12				Y			
XBP1	NM_005080	X-box binding protein 1				Y			
KLF4	NM_004235	Kruppel-like factor 4				Y			
CREBL1	X98054	cAMP responsive element BP-like 1	Y						
ID2	NM_002166	Inhibitor of DNA binding 2				Y			Y
ID4	AV646610	Inhibitor of DNA binding 4							Y
NR4A2	NM_006186	Nuclear receptor subfamily 4, group A, member 2				Y			
SON	NM_003103	SON DNA binding protein				Y			
DHX34	D50924	DEAD box polypeptide 34	Y						
HLF	NM_002126	Hepatic leukaemia factor				Y			
DDX1	NM_004939	DEAD/H (Asp-Glu-Ala-Asp/His) box polypeptide 1				Y			
MLL2	AF010403	Myeloid/lymphoid mixed lineage leukaemia 2	Y						
TCF8	NM_030751	Transcription factor 8				Y			
MEIS1	NM_002398	Meis1 (mouse) homologue				Y			
MEIS2	AF017418	Myeloid ecotropic viral integration site 1 homolog 2							Y
EGR1	NM_001964	Early growth response 1				Y			
MYBL2	X13293	Myeloblastosis viral oncogene homologue like 2	Y						
ATF3	NM_001674	Activating transcription factor 3				Y			Y
MAFK	AF059194	musculoaponeurotic fibrosarcoma oncogene	Y						
BTF3	NM_001207	Basic transcription factor 3				Y			
MSC	AF087036	Musculin	Y						
ATF7	X52943	Activating transcription factor 7	Y						
HOXA3	NM_153631	Homeobox A3						Y	
IRF1	NM_002198	Interferon regulatory factor 1						Y	
LOC152485	NM_178835	Hypothetical protein LOC152485						Y	
TGIF2	AB042646	TGFB-induced factor 2							Y
ELF4	NM_001421	E74-like factor 4							Y
NAB2	NM_005967	NGF1-A binding protein 2							

SUPPLEMENTAL TABLE 1 *continued*

Gene Symbol[†]	GenBank Accession number	Gene Description	GEA	DDD	Microarray studies				
					1	2	3	4	5
Protein Synthesis / ribosomal/ RNA splicing									
RBPMS	NM_006867	RNA-binding protein gene with multiple splicing				Y			
BRF2	NM_018310	Butyrate response factor 2				Y			
RPS25	NM_001028	Ribosomal protein S25				Y			
RPL10A	NM_007104	Ribosomal protein L10a				Y			
RPLP2	X56134	Ribosomal protein, large P2							Y
SRM160	NM_005839	Ser/Arg-related nuclear matrix				Y			
PCBP1	NM_006196	Poly(rC)-binding protein 1				Y			
SNURF	NM_003097	SNRPN upstream reading frame				Y			
TMEM30B	AL080078	cDNA DKFZp564D1462				Y			
MBNL1	NM_021038	KIAA0428 gene product				Y			
RANBP2L1	NM_005054	RAN binding protein2-like 1				Y			
RPL39	NM_001000	Ribosomal protein L39				Y			
RPL5	NM_000969	Ribosomal protein L5				Y			
RPL37A	BC016748	Ribosomal protein L37a		Y					
HNRPA1	NM_002136	Heterogeneous nuclear ribonucleoprotein A1				Y			
RPL37	D23661	Ribosomal protein L37		Y					
RPS19	BC018616	Ribosomal protein S19		Y		Y			
GRSF1	NM_002092	G-rich RNA sequence binding factor 1				Y			
RPL27A	BC020169	Ribosomal protein L27a		Y					
EEF1A1	NM_001402	Eukaryotic translation elongation factor 1 α1				Y			
Cell growth/ proliferation									
BHLHB2	AB004066	Basic helix loop helix domain containing class B2						Y	
PPAP2B	NM_003713	Phosphatidic acid phosphatase typbe 2b				Y			
GAS1	NM_002048	Growth arrest-specific 1				Y			
CLN5	NM_006493	Ceroid-lipofuscinosis, neuronal 5				Y			
FOSB	NM_006732	FBJ murine osteosarcoma viral oncogene homolog B				Y			
NGFB	NM_002506	Nerve growth factor, beta polypeptide				Y			
OS9	NM_006812	Amplified in osteosarcoma				Y			
SET	NM_003011	SET translcoation				Y			
ETS2	NM_005239	Avian erythroblastosis virus E26 oncogene homologue 2				Y			
EDF1	NM_003792	Endothelial differentiation-related factor 1				Y			
SKI	NM_003036	V-ski avian sarcoma viral oncogene homologue				Y			
ARHB	NM_004040	Ras homologue gene family, member B				Y			
S100A11	NM_005620	S100 calcium binding protein A11				Y			
HDGF	NM_004494	Hepatoma-derived gowth factor			Y				
AREG	M30704	Amphiregulin	Y						
SOCS3	BF514189	Supressor of cytokine signalling 3						Y	
DCBLD2	D29810	Endothelial cell derived neuropin like protein	Y						
TGFB3	J03241	Transforming growth factor beta					Y		
Insulin like growth factor system									
IGFBP5	NM_000599	Insulin-like growth factor binding protein 5				Y			
IGFBP3	NM_000598	Insulin-like growth factor binding protein 3				Y		Y	
PIK3R3	D88532	phosphoinositide-3-kinase, regulatory subunit	Y						

SUPPLEMENTAL TABLE 1 ***continued***

Gene Symbol[†]	GenBank Accession number	Gene Description	GEA	DDD	Microarray studies				
					1	2	3	4	5
Mitochondrial (Respiration/oxidation)									
GLUD1	NM_005271	Glutamate dehydrogenase 1				Y			
CYB5	NM_001914	Cytochrome b-5				Y			
NDUFA7	NM_005001	NADH dehydrogenase 1 alpha subcomplex				Y			
ATP5G3	NM_001689	ATP synthase, H+ transporting, mitochondrial F0				Y			
CYBA	M21186	Cytochrome B-245 alpha polypeptide	Y						
OAT	NM_000274	Ornithine aminotransferase				Y			Y
UQCRFS1	NM_006003	Ubiquinol-cytochrome c reductase, Rieske iron-suffer				Y			
SOD2	X07834	Manganese superoxide dismutase	Y						
CYP1B1	NM_000104	Cytochrome P450, subfamily 1				Y			Y
NDUFB5	NM_002492	NADH dehydrogenase 1 beta subcomplex				Y			
ACO2	NM_001098	Aconitase 2				Y			
GOT2	NM_002080	Glutamic-0xaloacetic transaminase 2				Y			
HCCS	NM_005333	Holocytochrome c synthase				Y			
ETFA	NM_000126	Electron transfer-flavoprotein, α polypeptide				Y			
ATP5F1	NM_001688	ATP synthase, H+ transporting, mitochondrial F0				Y			
OXA1L	NM_005015	Oxidase (cytochrom c) assembly like 1				Y			
Heat shock/ stress response/DNA damage response									
HSBP1	AF068754	Heat shock factor binding protein 1	Y						
GSR	X15722	Glutathione Reductase	Y						
DUSP5	NM_004419	Dual specificity phosphatase 5				Y			
GADD45A	NM_001924	Growth arrest and DNA-damage-inducible α							Y
GSTM1	J03817	Glutathione S transferase M1					Y		Y
MAPK14	L35253	p38 mitogen activated protein kinase	Y						
MAP4K1	U66464	Mitogen activated protein (kinase)4 1	Y						
Nervous system/neural									
GABARAP	NM_007278	GABA(A) receptor associated protein				Y			
CST3	NM_000099	Cystatin C				Y			
CFDP1	D85939	Craniofacial development protein 1	Y						
SYT5	X96783	Synaptotagmin V	Y						
ZFP103	NM_005667	Zinc finger protein homologous to Zfp103 in mouse				Y			
KIF5A	U06698	Neuronal kinesin heavy chain member 5A	Y						
PTGDS	AL045876	Prostaglandin D2 synthase 21 kDa (brain)						Y	
NEFH	AF203032	Neurofilament, heavy polypeptide 200kDa							Y
RAI14	AL050011	Retinoic Acid Induced 14	Y						
Serine/threonine kinsase									
CAMK4	D30742	Calcium/calmodulin portein kinase IV	Y						
PRKACA	NM_002730	Protein kinase cAMP-dependent, catalytic α				Y			
SDCCAG43	W73992	Sereologically defined colon cancer antigen 43							Y
Tyrosine kinase									
PTPRK	NM_002844	Protein tyrosine phosphatase, receptor type K				Y			
FGFR2	M87770	Fibroblast growth factor receptor 2						Y	
FGF2	NM_002006	Fibroblast growth factor 2 (basic)				Y			
PTK9	NM_002822	Protein tyrosine kinase 9				Y			

SUPPLEMENTAL TABLE 1 *continued*

Gene Symbol[†]	GenBank Accession number	Gene Description	GEA	DDD	Microarray studies				
					1	2	3	4	5
Nutritional response/metabolism/enzymatic synthesis									
PDK4	BF969355	Pyruvate dehydrogenase kinase, isoenzyme 4							Y
LDHB	Y00711	Lactate dehydrogenase B							Y
PP	NM_021129	Pyrophosphatase (inorganic)				Y			
HSD11B2	U26726	Hydroxysteroic (11-beta) dehydrogenase 2							Y
GNAI2	J03004	Guanine nucleotide binding protein, α inhibiting							Y
GALNAC4S-6ST	W91908	B cell RAG associated protein							Y
SORL1	Y08110	sortilin-related receptor containing LDLR class							Y
DHCR24	NM_014762	mRNA for KIAA0018 gene				Y			
CYP27A	M62401	Cytochrome P450, family 27, subfamily A, polypeptide 1							Y
ASS	AK027126	Argininosuccinate synthetase						Y	
MFGE8	NM_005928	Milk fat globule-EGF factor 8 protein				Y		Y	
ADH5	NM_000671	Alcohol dehydrogenase 5 (class III)				Y			
FACL1	NM_001995	Fatty-acid-coenzyme A ligase long-chain 1				Y			
ODC1	NM_002539	Ornithine decarboxylase 1				Y			
LGALS3	NM_002306	Lectin, galactoside-binding, soluble, 3							Y
SLC35A1	NM_006416	Solute family carrier 35				Y			
SENP1	AW965789	Sentrin/SUMO-specific protease							Y
STC2	AF098462	Stanniocalcin 2	Y						
PSPH	Y10275	Phosphoserine phosphatase	Y						
GYG	NM_004130	Glycogenin				Y			
Ion transport/small molecule transport									
RCV1	S43855	Recoverin	Y						
GPX3	NM_002084	Glutathione peroxidase 3 (plasma)						Y	
PDLIM1	NM020992	PDZ and Lim domain 1						Y	
FHL2	L42176	Four and a half LIM domains						Y	
CXCR4	L06797	Chemokine receptor 4	Y						
PTRX5	U49395	Ionotropic ATP receptor	Y						
CALU	NM_001219	Calumenin				Y			
SRI	NM_003130	Sorcin				Y			
SSR1	NM_003144	Signal sequence receptor, alpha				Y			
RCN2	NM_002902	Reticulocalbin 2				Y			
ATP2C1	NM_014382	ATPase, Ca++-sequestering				Y			
CACNG3	AF134640	Calcium channel, voltage dependent, γ subunit3						Y	
TM9SF2	NM_004800	Transmembrane 9 superfamily member 2				Y			
SGK	Y10032	Serum Glucocorticoid regulated kinase	Y			Y			
LTF	U07643	Lactotransferrin						Y	
SLC16A5	U59299	Solute carrier family 16						Y	
GABRB3	M82919	Gamma-aminobutynic acid A recpetor	Y						
KIP2	AB012955	DNA-dependent protein kinase catalytic subunit-interacting							Y
LAP3	NM_015907	Homo sapiens leucine aminopeptidase mRNA				Y			
ITPR2	NM_002223	Inositol 1,4,5-triphosphate receptor, type 2				Y			
Oncogenesis									
TACC1	NM_006283	Transforming, acidic coiled-coil containing protein 1				Y			

SUPPLEMENTAL TABLE 1 ***continued***

Gene Symbol[†]	GenBank Accession number	Gene Description	GEA	DDD	Microarray studies				
					1	2	3	4	5
Protease inhibitor									
SERPINB1	M93056	Serine (or cysteine) proteinase inhibitor, clade B member 1						Y	
SERPINB9	U71364	Serine proteinase inhibitor p19	Y						
WFDC2	X63187	WAP four-disulfide core domain 2						Y	
Phosphatase (Receptor)									
DUSP6	X93920	Dual specificity phosphatatse 6							Y
PTPRN	L18983	Tyrosine phosphatase receptor type N	Y						
Membrane traffiking/ cell signalling									
CD9	NM_001769	CD9 antigen (p24)				Y			
PHB2	NM_007273	REA B-cell associated protein				Y			
CXADR	NM_001338	Coxsackie virus and adenovirus receptor				Y			
LEPROT	NM_017526	mRNA for leptin receptor gene-related protein				Y			
DNAJB12	AL050381	DnaJ (Hsp40) homolgue subfamily B member 12	Y						
ITGA2B	J02764	Integrin alpha 2B			Y				
DBI	NM_020548	Acyl-CoA-BP			Y				
IL2RA	NM_000417	IL2 receptor alpha chain			Y				
CALM1	NM_001743	Calmodulin 1				Y			
TM9SF1	NM_006405	Transmembrane 9 superfamily member 1				Y			
ADAM10	NM_001110	A disintegrin and metalloprotease domain 10				Y			
LY6E	Z68179	Lymphocyte antigen 6 complex, locus E						Y	
SSR3	NM_007107	Signal sequence receptor, gamma				Y			
TLOC1	NM_003262	Translocation protein 1				Y			
B3GNT3	AB015630	Beta-1,3-N-acetylglucosaminyltransferase	Y						
SNX26	AA767103	Sorting Nexin 26	Y						
SRD5A1	NM_001047	Steroid-5-α-reductase, α polypeptide 1				Y			
GFRA3	AF051767	GDNF family receptor alpha 3	Y						
RAI3	NM_003979	Retinoic acid induced 3						Y	
PTGER4	L28175	Prostaglandin E receptor 4	Y						
TMEM50B	NM_006134	(C21orf4)/ transmemrane protein 50B				Y			
Signal Transduction									
IL6ST	M57230	IL-6 Signal transducer	Y						
RAB2	NM_004761	Member RAs oncogene family				Y			
GNB1	NM_002074	Guanine nucleotide binding protein β polypeptide				Y			
WIF1	AF122922	WNT inhibitory factor 1						Y	
OSBP	NM_002556	Oxysterol binding protein				Y			
SHB	NM_003028	Src homology 2 domain containing adaptor protein B			Y	Y			
Proteolysis									
CTSB	NM_001908	Cathepsin B				Y			Y
PSMA6	NM_087345	Proteasome subunit α type 6				Y			
PSMB7	NM_002799	Proteasome subunit β type, 7				Y			
CPE	NM_001873	Carboxypeptidase E				Y			
SPCS2	NM_014752	KIAA0102 protein				Y			
PSMA5	NM_002790	Proteasome subuit α type 5				Y			
ADAMTS7	NM_014272	Homo sapiens a disintegrin-like & metalloprotease				Y			
CTSH	NM_004390	Cathepsin H				Y			
PSMA3	NM_002788	Proteasome subunit α type 3				Y			

SUPPLEMENTAL TABLE 1 ***continued***

Gene Symbol[†]	GenBank Accession number	Gene Description	GEA	DDD	Microarray studies 1	2	3	4	5
Protein traffic/organisation									
ARCN1	NM_001655	Archain 1				Y			
PPP2CB	NM_004156	Protein phosphatase 2 catyltic subunit β				Y			
LMAN	NM_005570	Lectin, mannose binding 1				Y			
KDELR1	NM_006801	KDEL (lys-Asp-Glu-Leu) endoplasmic reticulum protein				Y			
MDG1	NM_012328	Microvascular endothelial differentiation gene 1				Y			
PPIC	NM_000943	Peptidylpropyl isomerase C				Y			
PDIR	NM_006810	For protein disulfide isomerase related				Y			
SEC61G	NM_014302	Sec61 gamma				Y			
YWHAZ	NM_003406	Tyrosine 3-monooxygenase				Y			
YWHAB	NM_003404	Tyrosine 3-monooxygenase				Y			
GRP58	NM_005313	Glucose regualted protein, 58kD				Y			
KDELR2	NM_006854	KDEL (Lys-Asp-Glu-Leu) endoplasmic reticulum protein				Y			
TRAM	NM_014294	Translocating chain-associating membrane protein				Y			
ARF1	NM_001658	ADP ribosylation factor 1				Y			
Tumour suppressor									
CAV2	NM_001233	Caveolin 2				Y			Y
PDGFRL	NM_006207	Platelet derived growth factor receptor like							
N33	NM_006765	Putative prostate cancer tumour suppressor				Y			
VCL	NM_003373	Vinculin				Y			
ST13	NM_003932	Supression of tumorigenicity 13				Y			
Unknown									
GEF-2	NM_007285	Ganglioside expression factor 2				Y			
SSFA2	NM_006751	Sperm specific antigen 2				Y			
GDI2	NM_001494	GDP dissociation inhibitor 2				Y			
EIF4A1	U79273	Human clone 23933 mRNA				Y			
GOLGB1	NM_004487	Golgi autoantigen, golgin superfamily b				Y			
AOE372	NM-006406	Thioredoxin peroxidase				Y			
GNG10	NM_004125	Guanine nucleotide binding protein 10				Y			
PBP	NM_002567	Prostatic binding protein				Y			
UAP1	NM_003115	UDP-N-acetylglucosamine pyrophosphorylase 1				Y			
TAGLN	NM_003186	Transgelin		Y					
C6ORF32	U49187	Chromsome 6 open reading frame 32	Y						
MXRA5	AF245505	DKFZp564I1922 Adlican						Y	
APCDD1	AI827230	Adenomatosis polposis coli down-regulated 1							Y
APM2	BF965257	Adipose specific 2							Y
ARID4B	W84531	RBP1-like	Y						
CKIP-1	AK021865	CK2 interacting protein 1							Y
ESTs									
	AA632025							Y	
	AI017213	EST weakly similar to N21_nuclear envelope	Y						

SUPPLEMENTAL TABLE 1 ***continued***

Gene Symbol[†]	GenBank Accession number	Gene Description	GEA	DDD	Microarray studies 1	2	3	4	5
Hypothetical Protein									
KIAA0438	NM_014819					Y			
KIAA0776	XM_035970					Y			
KIAA0089	XM_046056					Y			
KIAA0193	NM_014766					Y			
KIAA0747	NM_015292					Y			
ANAPC13	XM_098173					Y			
KIAA0697	XM_036687					Y			
KIAA0544	XM_048119					Y			
KIAA0539	AB01111		Y						
KIAA0989	NM_016201					Y			
KIAA0056	XM_027479					Y			
FLJ12671	AL096734		Y						
KIAA0068	XM_039225					Y			

[†]Official Gene Symbol from the HUGO Gene Nomenclature Committee. Abbreviations: GEA: Gene Expression Atlas DDD: Digital Differential Display. Microarray studies: 1. Bubendorf *et al* 1999, 2. Dhanaskeran *et al*, 2001, 3. Chetcuti et al, 2001, 4. & 5. Ashida et al, 2004 (4. down-regulated in prostate cancer & HGPIN, 5. Down-regulated in transition from HGPIN to prostate cancer).
